# Washable and Reliable Textile Electrodes Embedded into Underwear Fabric for Electrocardiography (ECG) Monitoring

**DOI:** 10.3390/ma11020256

**Published:** 2018-02-07

**Authors:** Amale Ankhili, Xuyuan Tao, Cédric Cochrane, David Coulon, Vladan Koncar

**Affiliations:** 1 École Nationale Supérieure des Arts et Industries Textiles/Génie et Matériaux Textiles Laboratory (ENSAIT/GEMTEX), 2 Allée Louis et Victor Champier, F-59100 Roubaix, France; xuyuan.tao@ensait.fr (X.T.); cedric.cochrane@ensait.fr (C.C.); vladan.koncar@ensait.fr (V.K.); 2 GEMTEX, University of Lille, Cité Scientifique, F-59650 Villeneuve d’Ascq, France; 3 @HEALTH, Europarc de Pichaury, 1330 Rue Jean René Guillibert Gauthier de la Lauzière, F-13290 Aix-en-Provence, France; dcoulon@healthcardionexion.com

**Keywords:** textile electrodes, ECG, electrical properties, washability

## Abstract

A medical quality electrocardiogram (ECG) signal is necessary for permanent monitoring, and an accurate heart examination can be obtained from instrumented underwear only if it is equipped with high-quality, flexible, textile-based electrodes guaranteeing low contact resistance with the skin. The main objective of this article is to develop reliable and washable ECG monitoring underwear able to record and wirelessly send an ECG signal in real time to a smart phone and further to a cloud. The article focuses on textile electrode design and production guaranteeing optimal contact impedance. Therefore, different types of textile fabrics were coated with modified poly(3,4-ethylenedioxythiophene):poly(styrenesulfonate) (PEDOT:PSS) in order to develop and manufacture reliable and washable textile electrodes assembled to female underwear (bras), by sewing using commercially available conductive yarns. Washability tests of connected underwear containing textile electrodes and conductive threads were carried out up to 50 washing cycles. The influence of standardized washing cycles on the quality of ECG signals and the electrical properties of the textile electrodes were investigated and characterized.

## 1. Introduction

The improvement of human health is an important objective that remains a relevant aim of research. Currently, cardiovascular diseases are the first cause of lethal issues worldwide. The “@Health” company has been created to improve health conditions by revolutionizing the way in which cardiovascular problems are diagnosed. The purpose of the company’s approach is not only to meet the needs for pre-diagnostic development linked to diseases whose symptoms only appear too late (or not at all), but also to increase economic requirements, as well as the need for mass prevention policy design.

As cardiovascular diseases provoke 30% of deaths worldwide, @Health is developing custom medical monitoring solutions to anticipate the onset of symptoms and to implement as quickly as possible a treatment that will increase the chance of recovery. 

Analysis and alerting using real-time electrocardiogram (ECG) signals could be considered as a possible solution to decrease the death toll caused by heart diseases. The ECG signal quality is essential for permanent and accurate heart monitoring. It can be obtained from high-quality, flexible, textile-based electrodes embedded into underwear [1]. These electrodes should guarantee permanent contact with the skin, optimal reliability, washability, and acceptable lifetime.

A number of more or less flexible and rarely stretchable electrodes already exist on the market or as prototypes in research laboratories. However, they are not yet adapted to reliable and washable underwear for ECG monitoring in real time able to generate a medical quality signal. The most-used electrodes are based on silver–silver chloride (Ag/AgCl). They consist of a silver base with an ionic compound silver chloride layer on its surface [2,3]. Nevertheless, they can provoke cutaneous reactions in combination with the hydrogel that is used to improve the contact impedance, after prolonged contact with the skin [4]. Furthermore, after repeated use, the silver chloride film can be damaged due to mechanical stresses. This type of electrode can be used for short-term diagnostic recording such as taking a clinical electrocardiogram in the hospital [3]. The basic metal plate (inflexible and non-stretchable) electrode is also used for ECG measurement. It contains a metallic conductor with a thin layer of electrolyte gel between the metal and the skin to insure a good contact with the skin and to decrease the contact resistance [3]. These electrodes could also be made of metallic foils. Sometimes they are produced in the form of a suction electrode. Metals used for this type of electrode include silver, gold, platinum, and nickel-silver alloys [3].

Thin-film flexible electrodes are basically the same as metallic plate electrodes; the only difference is the thickness of the metal, which is less than a micron. These metal films are usually supported on a flexible plastic substrate, such as polyester or polyimide plate [1,3].

Textile substrates intended to be used within underwear are expected to be lightweight, flexible, stretchable, conformable, washable, and long-lasting [5,6]. Conductive patterns on textiles can be achieved by micro-contact embedded electrodes, inkjet printing, and screen printing [1]. Thin stainless steel, copper, or other metal wires are employed to sew conductive patterns on textile fabrics by embroidery [7]. On the other side, poly(3,4-ethylenedioxythiophene):poly(styrenesulfonate) (PEDOT:PSS) has exhibited growing potential, not only in flexible electronics, but also in biomedical devices, due to its excellent electrical properties, environmental stability, and decent biocompatibility [8,9]. Electrodes made of PEDOT:PSS have already been successfully used in ECG measurements [6]. 

In this study, the fabrication of fully wearable electrodes using dip-coating technology of conducting polymer PEDOT:PSS on a commercial knitted fabric is reported. Knitted fabrics were chosen as substrates because of their high level of stretchability, allowing a good contact with the skin. Textile fabrics coated with PEDOT:PSS were assembled into textiles structures, such as bras, by sewing with conductive yarn, then textile electrodes were connected to ab ECG measurement device and high-quality ECG signals were successfully recorded. Washability tests of connected underwear were carried out up to 50 washing cycles, and ECG data, recorded from a healthy volunteer, were found to be stable. This experiment paves the way for the fabrication of low cost electrodes for cutaneous electrophysiology. The influence of the encapsulation process on the electrical properties of electronic modules during washing tests is under development in our laboratory [10] and will be investigated and analyzed later on. 

## 2. Materials and Methods

Poly(3,4-ethylenedioxythiophene):poly(styrenesulfonate), also called PEDOT:PSS, type Clevios was purchased from Heraeus Conductive Polymers Division (Hanau, Germany). The conductive thread employed (Shieldtex 234/34-2 ply HCB) is silver-coated polyamide yarn, which is used for signal transmission. The linear resistance is less than 100 Ω/m, which is acceptable for sensing an ECG signal in a low energy consumption system. For the electrical surface resistance measurements, a Keithley 8009 device (Keithley Instruments Sarl, Les Ulis, France) was used. The washing tests were performed according to the standard ISO 6330 [11]. The washing test laboratory machine was a Datacolor AHIBA IR (Paris, France).

For ECG measurements, an ambulatory device “Colson CardiPocket 2” (Cuers, France) was connected to textile-based PEDOT:PSS electrodes in order to determine the quality of the ECG signal.

Three textile electrodes were manufactured by dip-coating. Polyamide, polyester, and cotton knitted fabrics were soaked in a chemically modified PEDOT:PSS solution and then dried at 110 °C for 1 h. The electrodes have a solid circle shape with a diameter of 11 cm. These circular electrodes are cut to small electrodes (15 cm^2^) for integration into bras for women. The commercially available PEDOT:PSS solution had to be chemically modified in order to make it suitable for each textile fabric for the dip-coating process, particularly to guarantee optimal washing behavior. The nature of the chemical modification cannot be revealed in this article because of confidentiality issues.

After drying, electrodes were rinsed in distillated water to eliminate all remaining particles. 

## 3. Results and Discussion

### 3.1. PEDOT:PSS Absorption

Textile fabrics exhibit various features depending on the structure and type of fibers. For this reason, three different knitted fabrics were selected to make ECG electrodes. Textiles need to be coated not only on the surface of the knitted structure, but also inside, providing continuous contact between the yarns during its mechanical deformation. The viscoelastic properties of PEDOT:PSS formulation allows a homogeneous coating of flexible knitted textiles, in our case, cotton, polyamide, and polyester. Table 1 shows that the cotton electrode absorbs more PEDOT:PSS than the polyamide or polyester electrodes. This is probably due to different adhesions in the function of the substrate material, particularly for polyester. This will be verified in our future studies.

### 3.2. Electrical Characterization

**Figure 1:**
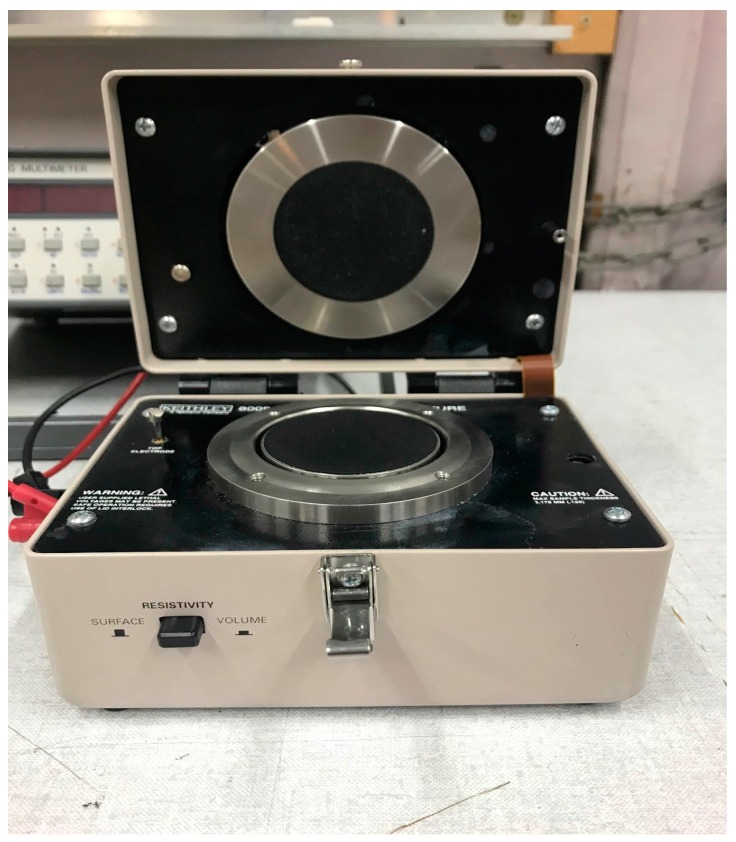
Photo of KEITHLEY 8009 resistivity test fixture.

The experimental characterization was realized according to ASTM D 257-99 [12] and IEC 61340-5-1 Standards [13]. Surface resistivity is defined as the electrical resistance of the surface of the material. It is measured from electrode to electrode along the surface of the sample. Surface resistivity measurement is carried out by a KEITHLEY 8009 resistivity test fixture (Figure 1). The sample was placed between two concentric ring electrodes. Its resistance was measured by sourcing a known voltage, and measuring the resulting current using Ohm’s Law. From the resistance measurement, the resistivity was determined based on the physical dimensions of the test sample (Equation (1)). A lower value of surface resistivity allows a good acquisition of the ECG signal, even if it is not directly related to the contact impedance between the electrode and the skin.

(1) $$\rho s=53.4\frac{V}{I}$$

where ρs is the surface resistivity of the sample, *V* is the applied voltage from the electrometer, and *I* is the current reading from the electrometer.

Table 2 shows that the average surface resistivity of the cotton electrode (21.02 kΩ) is lower than that of the other electrodes. The difference in electrical conductivity between these electrodes seems to result from the PEDOT:PSS coating. As more PEDOT:PSS was absorbed by cotton, the cotton electrode consequently has the best electrical properties, followed by the polyamide and finally the polyester electrodes. To improve the absorption of PEDOT:PSS by hydrophobic substrates, the fabric surface could be treated by atmospheric plasma. In our case, there was not any surface preparation. 

The washability issue is always an obstacle in terms of application, reducing the reliability of wearable electrodes, and therefore making them not ready for the market and use in real life. Due to the capillary effect, textile substrates absorb water and the mechanical stresses provoked by the washing process may destroy the electrical contacts between the conductive threads. As a result, the electrical resistance becomes uncontrollable after several washing cycles and the wearable electrodes become unstable and, in some cases, stop functioning.

To evaluate the electrical performance of our textile electrodes in a wearable configuration for long-term monitoring, 50 washing cycles at 40 °C for 30 min were realized according to ISO 6330 [11]. The washing standards offer a variety of water temperature levels for different applications, ranging from 30 to 92 °C. In this study, the rotation speed is 30 rpm. Surface resistivity is measured after every washing cycle. Then ratio R**_i_**/R**_0_** is calculated, where R**_0_** is the surface resistance before washing and R**_i_** is the surface resistance after the i-th washing cycle.

**Figure 2:**
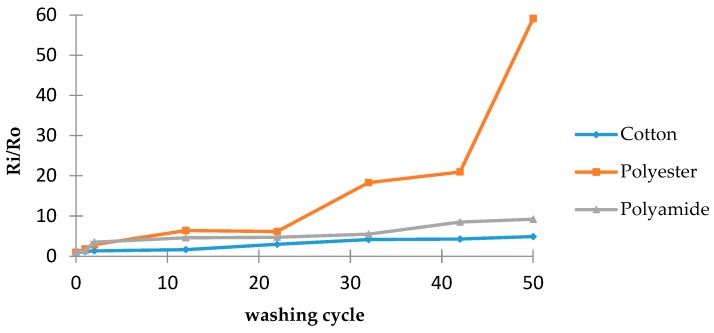
The evolution of R**_i_**/R**_0_** versus washing cycle.

Figure 2 shows the evolution of surface resistance values of textile electrodes during washing tests. For cotton- and polyamide-based electrodes, the resistance increases linearly with the number of washing cycle. After the 50th washing cycle, this increase for cotton electrodes (4.9 times) is slower than that for polyamide (9.2 times) or polyester ones (59 times). This result can be explained by the fact that polyester does not absorb PEDOT:PSS as well as cotton or polyamide. Otherwise, we can assume that polyester will exhibit the same linear behavior but with a different slope. At the same time, we can also assume the remaining quantity of PEDOT:PSS after 40 cycles leads to improper conduction, hence a sudden R**_i_** measurement increase, which indicates that PEDOT:PSS is evenly covered on the surface and consumed faster during washing cycles for polyester than for cotton or polyamide because of the poor adhesion of PEDOT:PSS to polyester. It should be mentioned that this failure of the coating derives from the mechanical movement (bending, friction, etc.) during the washing.

### 3.3. Evaluation of Textile Electrodes in ECG Monitoring

An ECG signal of medical quality is necessary for permanent monitoring, and accurate heart examining can be obtained from instrumented underwear only if it is equipped with high-quality, flexible, textile-based electrodes guaranteeing low contact impedance with the skin. In order to assess the performance of our developed textile electrodes in biomedical monitoring in depth, we investigated electrocardiography recordings before and after washing as well as during motion. 

**Figure 3:**
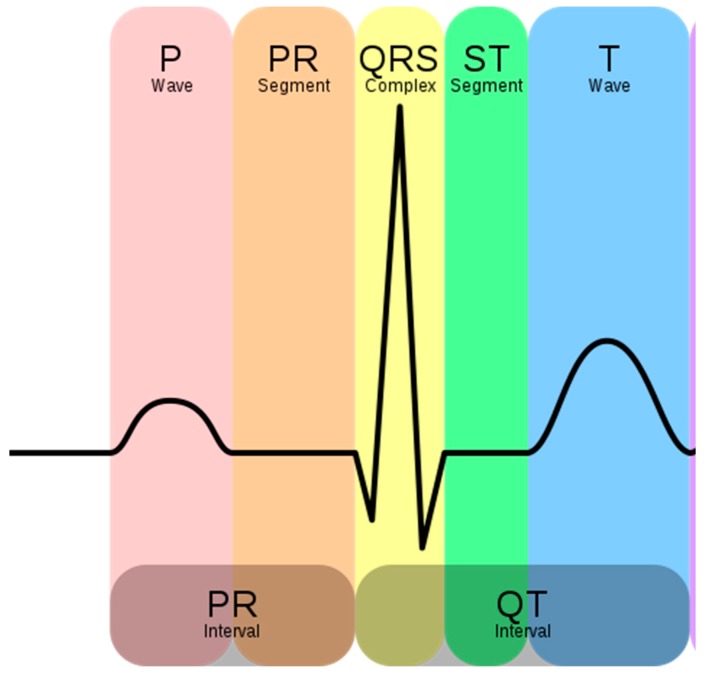
Schematic representation of a normal electrocardiogram (ECG). P, Q, R, S, T are graphical deflections of a typical electrocardiogram. They express depolarization and repolarization of the human heart.

Figure 3 shows the schematic representation of a normal ECG. The P wave represents atrial depolarization; the QRS (QRS complex corresponds to a specific part of the ECG considering Q, R, and S waves; It corresponds to the depolarization of the right and left ventricles of the human heart) complex represents ventricular depolarization, and the T wave represents ventricular repolarization.

ECGs are normally printed on a grid. The horizontal axis represents time and the vertical axis represents voltage. In an ECG signal, Leads I, II, and III are called the limb leads. The electrodes that form these signals are located on the limbs—one on each arm and one on the left leg [14,15,16]. The limb leads form the points of what is known as Einthoven’s triangle [17]: 

Lead I: the voltage between the left arm (LA) electrode and right arm (RA) electrode.

Lead II: the voltage between the left leg (LL) electrode and the right arm (RA) electrode.

Lead III: the voltage between the left leg (LL) electrode and the left arm (LA) electrode. 

**Figure 4:**
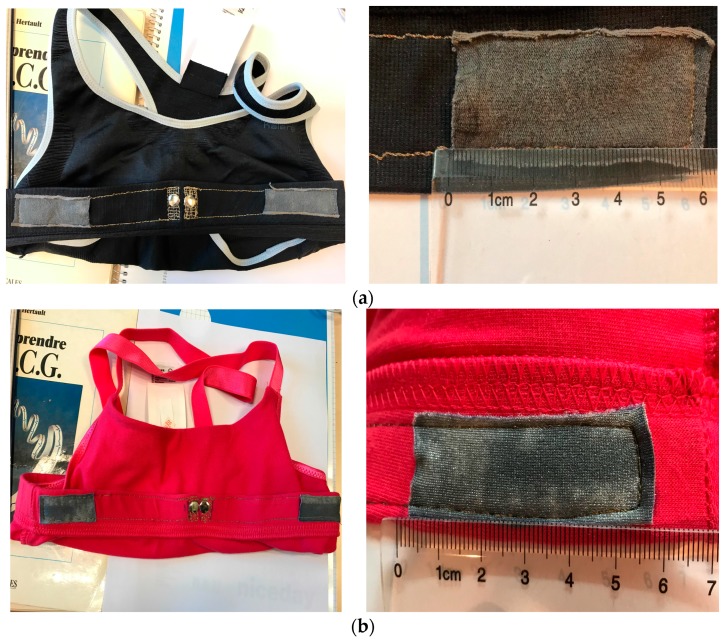
(**a**) Cotton electrodes sewn into bras; (**b**) Polyamide electrodes sewn into bras.

Textile electrodes were sewn into bras with conductive yarns, in order to connect them to the ECG device by using snap fastener buttons with crocodile clips (Figure 4). The distance between the electrodes is 14 cm. The size of electrodes is 15 cm^2^ per electrode. When wearing the bra, the right arm is replaced by the right chest and the left arm by the left chest. In the right and left leg, we connect metallic medical electrodes; therefore, the performance of textile electrodes is more relevant in the first lead (lead I) because it represents the voltage between two sewn textile electrodes.

**Figure 5:**
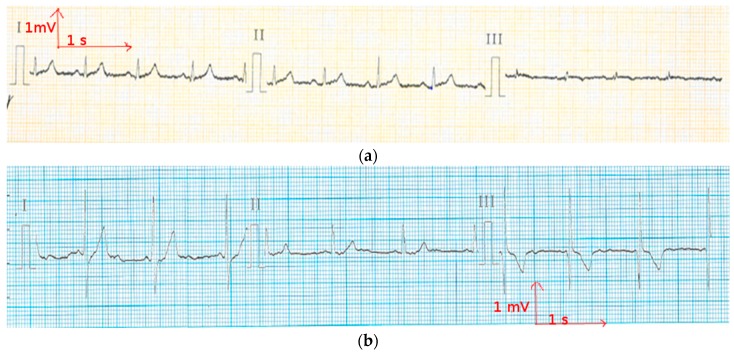
Electrocardiography at rest from the cotton electrode (**a**) before washing; and (**b**) after 50 washing cycles.

**Figure 6:**
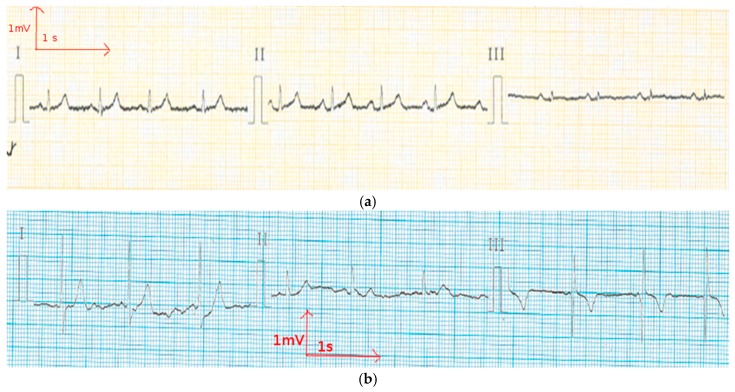
Electrocardiography at rest from the polyamide electrode (**a**) before washing; and (**b**) after 50 washing cycles.

**Figure 7:**
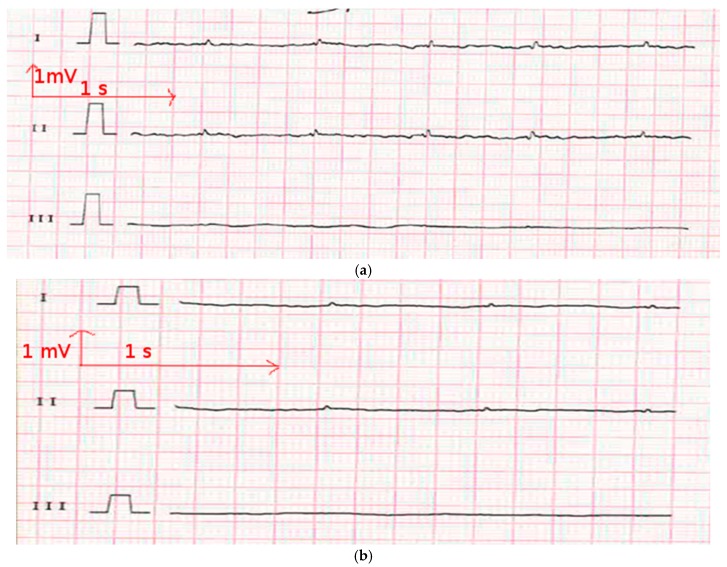
Electrocardiography at rest from the polyester electrode (**a**) before washing; and (**b**) after 50 washing cycles.

In order to reduce muscle movements and respiratory artifacts, as a first measurement, the volunteer was sitting at rest. This position allows obtaining high-quality signals that can be used to detect heart function anomalies. The above ECG measurement tests conducted for static postures were recorded successfully with no prior skin preparation or application of conductive gels. With the cotton and polyamide electrodes, decent ECG signals were obtained for medical analysis. Figure 5 and Figure 6 show that before and after 50 washing cycles, the P and Q wave and QRS complex corresponding to the different phases of polarization and depolarization of cardiac cells were easily observed in the signals recorded using both the cotton and polyamide electrodes. The ST (the ST segment is the interval between S and T waves; it is important to have a clear view of it to track ischemic issues) segment was also observed, which is very important for myocardial ischemia and ventricular arrhythmias detection. However, for the polyester-based PEDOT:PSS-coated electrodes (Figure 7), P, QRS, and T waves were not recognizable; therefore, the acquired signals from the polyester electrodes were not acceptable for our application. This failure is due not only to the lower quantity of PEDOT:PSS absorbed by polyester, but also to the high contact impedance between the skin and the polyester electrode. 

ECG signals were evaluated after 50 washing cycles, captured by the cotton and polyamide electrodes in motion to investigate if these electrodes are proper for long-term monitoring where no skin preparation is allowed and no gel is added. ECG measurements were conducted in three different postures: sitting on a chair with arms on a desk, walking, and climbing stairs. Good-quality signals should be obtained from a woman wearing a bra containing textile electrodes as she goes through her daily routine.

**Figure 8:**
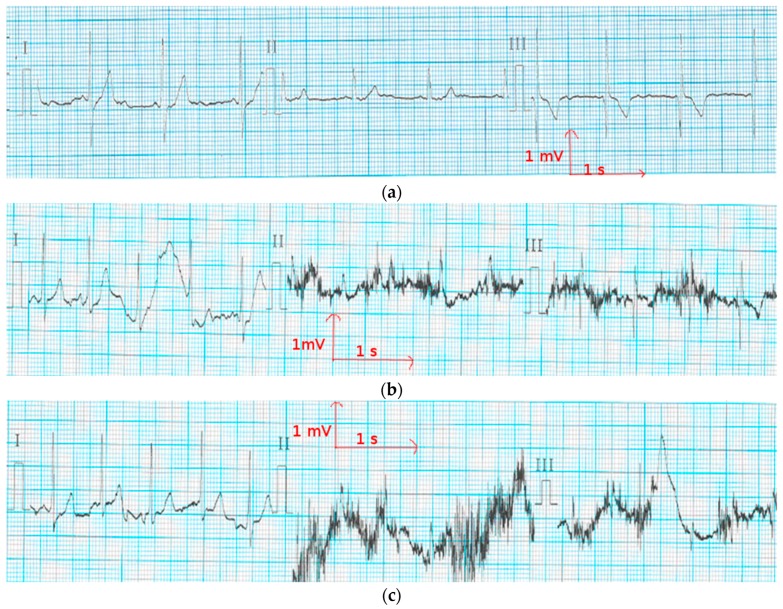
Electrocardiography from the polyamide electrodes after 50 washing cycles (**a**) at rest; (**b**) when walking; and (**c**) when climbing stairs.

**Figure 9:**
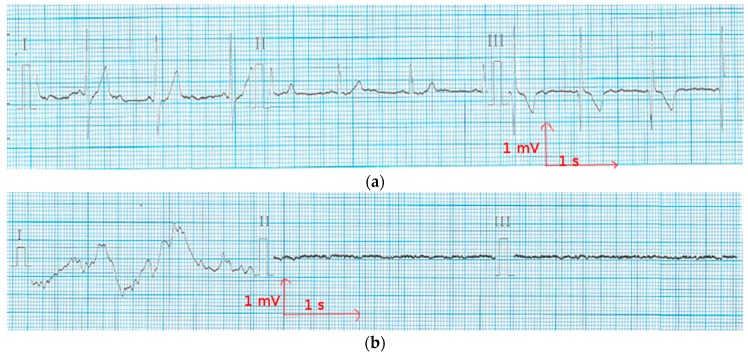

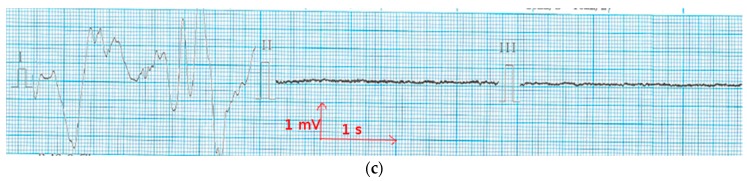
Electrocardiography from the cotton electrodes after 50 washing cycles (**a**) at rest; (**b**) when walking; and (**c**) when climbing stairs.

In ECG measurement, the quality of ECG signals depends on the contact impedance between the body and the wearable electrodes. This contact is liable to drop because of a change in motion or posture. In Figure 8 and Figure 9, there are three ECG signals recorded (a) at rest, (b) walking, and (c) climbing stairs. In all of the cases, lead I is relative to the signal recorded from the two chest electrodes, lead II between the right chest electrode and the left leg electrode, and lead III between the left chest electrode and the left leg electrode. It is obvious that the signal quality is best when only the two chest electrodes are used, particularly in motion. This case is the best for our application, as the ECG signal should be recorded only using the underwear. As it can be seen from Figure 8, using polyamide electrodes subjected to 50 washing cycles, the lead I of ECGs taken with the polyamide electrodes have stable P, R, and T wave amplitudes, indicating that the influence of motion is significantly lower on the recordings of these electrodes. Leads II and III exhibit some variation in amplitudes and are more contaminated by noise because, while walking and climbing stairs, the crocodile clip connected to the metallic medical electrodes on the legs tends to move. However, P, QRS, and T waves are still recognizable in lead I. Thus, the acquired signals are acceptable for our application. Figure 9 shows that with the cotton electrodes put through 50 washing cycles, at rest, the signal is quite good. However, during motion tests, the signals are not acceptable as P, QRS complex, and T waves are not detectable. This failure is due to the poor connection between the crocodile clips and the ECG device. We can also conclude that the surface resistivity of the electrode does not play an important role for the ECG recording, because after the washing process, the cotton electrodes with lower surface resistivity had worse ECG signals compared to the polyamide electrodes. We suppose that the contact impedance between the electrode and the skin is another factor that impacts the ECG recording.

## 4. Conclusions

In this paper, a chemical formulation has been developed, based on PEDOT:PSS appropriate for the dipping of various textile fabrics (cotton, polyamide, and polyester), making flexible electrodes for long-term monitoring as compared to gel electrodes (Ag/AgCl) which are disposable and cause skin irritation. There are three main advantages of the proposed solution in our paper over other conductive fabrics. Firstly, compared with those non-conductive textile fabrics, PEDOT:PSS-coated textile fabrics have similar mechanical properties, such as bending, stiffness, and abrasion [18]. (PEDOT:PSS). This is not the case for metallic-based electrodes which may be uncomfortable, such as medical Ag/AgCl ECG-gel-electrodes and metal clips. The wearer found that the textile electrodes are much more comfortable than metallic electrodes. Secondly, compared with traditional organic conductor-based conductive fabrics, the main drawbacks of poor washability and aging were solved within our approach to the PEDOT:PSS-coated fabric. Thirdly, the dip-coating technique can be easily applied during textile manufacturing and is fully compatible with knitted fabrics thanks to the rheological properties of PEDOT:PSS.

To evaluate the overall quality of the acquired ECG signals by textile electrodes, washability tests of underwear equipped with fully textile electrodes (cotton, polyamide, and polyester coated with PEDOT:PSS) connected to a “Colson CardiPocket 2” ECG recording device using conductive thread Shieldtex 234/34-2 ply HC+B were carried out up to 50 washing cycles. Several observations, important for smart and connected clothing development, have been noted: (i) the absorption of PEDOT:PSS depends on the nature of the intrinsic fibers of the textile used as a substrate; (ii) a commercially available solution with dispersed PEDOT:PSS Clevios is well adapted to the dip-coating of textile substrates; (iii) the obtained textile electrodes with cotton and polyamide as substrates are washable for at least 50 washing cycles and are able to generate a decent ECG signal, even in motion in the case of the polyamide electrodes. This may be explained by the partial dyeing of the textile fibers with the PEDOT:PSS solution, as opposed to just a coating, highlighting the long-term stability of the electrodes.

It is very important to underline the fact that the skin condition will vary from person to person. The quality and the shape of the ECG signal depends not only on the surface resistivity of electrodes but also on the contact impedance between the skin and electrodes. For this reason, our future research work will focus on a novel method to optimize the ECG signal acquired by our electrodes. This method will be based on minimizing the contact impedance in order to analyze how it varies as a function of the type of textile electrodes and the skin properties of the wearer. 

## Figures and Tables

**Table 1:** Mass weight percentage of PEDOT:PSS after dip-coating.

Textile Electrodes	Percentage of Absorbed PEDOT:PSS after Drying (wt %)
Cotton	6%
Polyamide	4.4%
Polyester	3.2%

**Table 2:** Surface resistivity (kΩ) as a function of the type of the substrate coated by PEDOT:PSS.

Textile Electrodes	Surface Resistivity (kΩ)	Standard Deviation (kΩ)
Cotton	21.02	0.64
Polyamide	34.26	0.74
Polyester	36.31	0.84
